# Oral amyloidosis: an update

**DOI:** 10.4317/medoral.25761

**Published:** 2023-06-18

**Authors:** Flávia Sirotheau Corrêa Pontes, Gabrielle Bastos Machado Ferreira, Felipe Paiva Fonseca, Tatiana Foscaldo Ribeiro Abreu Ribeiro, Patrícia Carlos Caldeira, Thalita Soares Tavares, Ana Paula Guerreiro Bentes, Márcio Ajudarte Lopes, Tarcília Aparecida da Silva, Jeanne Gisele Rodrigues de Lemos, Hélder Antônio Rebelo Pontes

**Affiliations:** 1 Professor. Service of Oral Pathology, João de Barros Barreto University Hospital, Federal University of Pará, Belém, Brazil; 2 Graduate student, Oral Pathology Department, João de Barros Barreto University Hospital/Universidade Federal do Para, Belém, Pará, Brazil; 3 Professor. Department of Oral Surgery, Pathology and Clinical Dentistry, School of Dentistry, Universidade Federal de Minas Gerais, Belo Horizonte, Minas Gerais, Brazil; 4 Professor. Analytical Laboratory of Restorative Biomaterials (LABiom-R), School of Dentistry, Federal Fluminense University, Niterói, Rio de Janeiro, Brazil; 5 PHD. Department of Oral Diagnosis, Piracicaba Dental School, University of Campinas (UNICAMP), Piracicaba, SP, Brazil

## Abstract

**Background:**

Amyloidosis is a disease characterized by the progressive deposition of abnormal proteins that can occur in any organ. In the oral cavity, the tongue is the most common affected site, usually causing macroglossia. Biopsy is essential for the diagnosis and the occurrence of its systemic form is mandatory to be investigated. This systematic review evaluated the existing information in the literature on Amyloidosis in the oral cavity to allow a more comprehensive and updated analysis of its clinicopathological characteristics, as well as to explore the main forms of treatment and prognostic factors.

**Material and Methods:**

Electronic searches were undertaken in five databases supplemented by manual scrutiny.

**Results:**

A total of 111 studies were included with 158 individuals.

**Conclusions:**

The disease had a higher prevalence in women, the tongue was the most affected site, as well as the systemic form of the disease. The worst prognosis was for cases of systemic amyloidosis associated with multiple myeloma.

** Key words:**Amyloidosis, mouth, Congo red.

## Introduction

Amyloidosis is a group of diseases characterized by abnormal and progressive extracellular deposition of insoluble fibrillar proteins, which can occur in any organ. Amyloidosis can be systemic or localized, primary or secondary, acquired or hereditary. Systemic amyloidosis occurs when it affects different organs, while localized amyloidosis when it affects a single organ. The primary or secondary classification is related to the presence or absence of a pre-existing pathology and the classification as acquired or hereditary is related to the nature of the origin of the deposited proteins. (1,2,3).

Oral involvement is usually secondary to systemic amyloidosis. Tongue and buccal mucosa are the most affected sites, with a common presentation of macroglossia, enlarged and hardened tongue (3,4,5). The histopathological analysis using Congo red staining is considered the gold standard for diagnosis because fibrillar protein deposits show green-yellow birefringence on polarized light (5,6).

Treatment depends on the type of amyloidosis, whether there are associated diseases and the systemic condition of patient and ranges from surgical resection, chemotherapy, and organ and hematopoietic stem cells transplantation (1). The prognosis depends on the condition of the affected organ, but localized amyloidosis appears to have a better prognosis compared to systemic amyloidosis (5,7).

The purpose of the current systematic review was to evaluate the existing information in the literature on Amyloidosis manifestation in the oral cavity to allow a more comprehensive and updated analysis of its clinicopathological characteristics, as well as to explore treatment and prognosis.

## Material and Methods

- Search strategies

**Figure 1:**
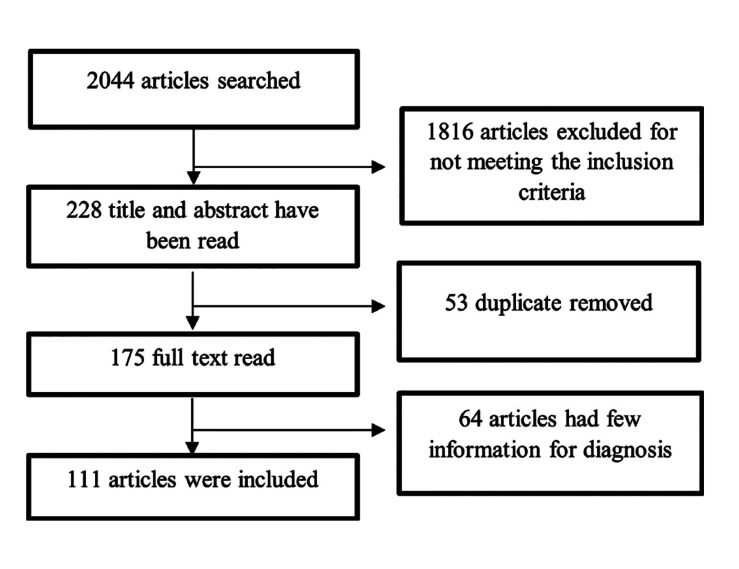
Flow chart of searching process.

An electronic search without time restrictions was performed until May 2022 in the following databases: PubMed (National Library of Medicine), Embase (Elsevier), Science Direct (Elsevier), Scopus (Elsevier), and Web of Science (Clarivate Analytics). Search schemes employed in databases combined the terms "amyloidosis” AND “oral cavity” OR “parotid” OR “submandibular". Titles and abstracts were analyzed for the selection of articles. A manual search of all journals related to oral amyloidosis was performed (Fig. 1).

- Inclusion and exclusion criteria

**Figure 2:**
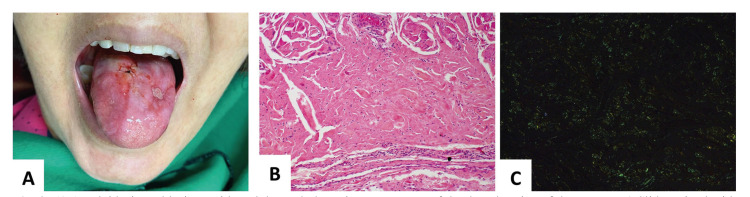
A) Amyloidosis oral lesions with nodular and ulcerative appearance of the dorsal region of the tongue. B) Slide stained with hematoxylin & eosin showing an amount of eosinophilic fibrillar material, suggestive of amyloid. (H&E, 10X). C) Slide stained with Congo Red showing yellow-green birefringence in polarized light for confirmation of amyloidosis (Congo Red, 10X).

Eligibility criteria included publications reporting cases or case series of amyloidosis in the oral cavity. The articles needed to present clinical and histopathological features associated with the manifestations of the disease in the oral cavity. Congo red staining on biopsy and green-yellow birefringence on polarized light microscopy were used to confirm the diagnosis of cases of amyloidosis (Fig. 2). Exclusion criteria were articles in languages other than English or Portuguese, immunohistochemical studies, radiological studies, histopathological studies, cytological studies, animal studies and review articles, unless any of these publication categories reported some case with enough diagnostic and clinicopathological information.

- Study selection

The titles and abstracts of all studies found through the electronic search were read by two authors (HARP and GBMF) to confirm the inclusion criteria. The full text of articles without sufficient information in the titles/abstracts was retrieved to assist the two authors in inclusion or exclusion. In the second step, two authors assessed the full texts and those meeting the eligibility criteria were included. Discrepancies were resolved by consensus or in a consultation with two senior authors (FSCP and HARP).

- Data extraction

Data extraction was performed using a specially designed Table. For each of the report included, the following data were extracted on a standard Table, when available: author, year of publication, country of publication, patients’ sex, age, evolution time (months), lesion size in millimeters (mm), lesion location, clinical appearance, disease form (localized or systemic), type of occurrence (primary or secondary), oral clinical symptoms, systemic clinical symptoms, presence of other organ affected, diagnostic resources (radiography, computed tomography, magnetic resonance imaging, ultrasound), histological aspects, associated disease, treatment, recurrence, and follow-up time. Lesion size was determined according to the largest diameter reported in the publications.

## Results

The total search for studies presented 2044 articles, 23 articles found in the Scopus database, 564 articles found in Embase, 818 in Web of Science, 65 in SCIENCE DIRECT, and 574 in PubMed. Of the total of studies (2044 articles), 1816 were excluded for not presenting cases of oral amyloidosis, resulting in 228 studies. Of the 228, 53 represented duplicate articles in more than one database. The full texts of the 175 studies were read and evaluated which led to the exclusion of 64 studies for not reporting sufficient clinical and histological information that could confirm the diagnosis of amyloidosis. Thus, 111 studies and 158 cases were included in the review. The clinical and demographic data are shown in Table 1.

**Figure 3:**
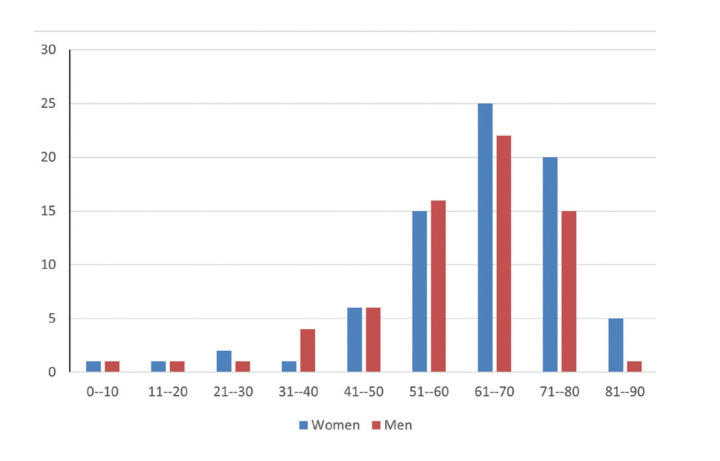
Distribution of cases of amyloidosis in the oral cavity considering sex and age of individuals.

Regarding the prevalence between the sexes, the lesion had a slightly higher prevalence in women than in men, with a male:famale ratio of 1.22:1. The mean age of patients was 61.1 years (range 7-90 years); women were older (mean age 62.5 years [range 10-90 years]) than men (mean age 59.4 years [range 7-82 years]). Fig. 3 shows the distribution of lesions according to the age group and sex, with a higher prevalence in the fifth to eighth decades of life. Patients had a mean disease course time of 11.4 months (range 1-48 months).

The most reported clinical appearance were nodular swelling on the tongue, macroglossia, edema in the affected region, ulcerative lesions, purplish/hemorrhagic or whitish/yellow papular lesions. Fig. 2 shows a case of nodular and ulcerative amyloidosis affecting the tongue.

**Table 1:**
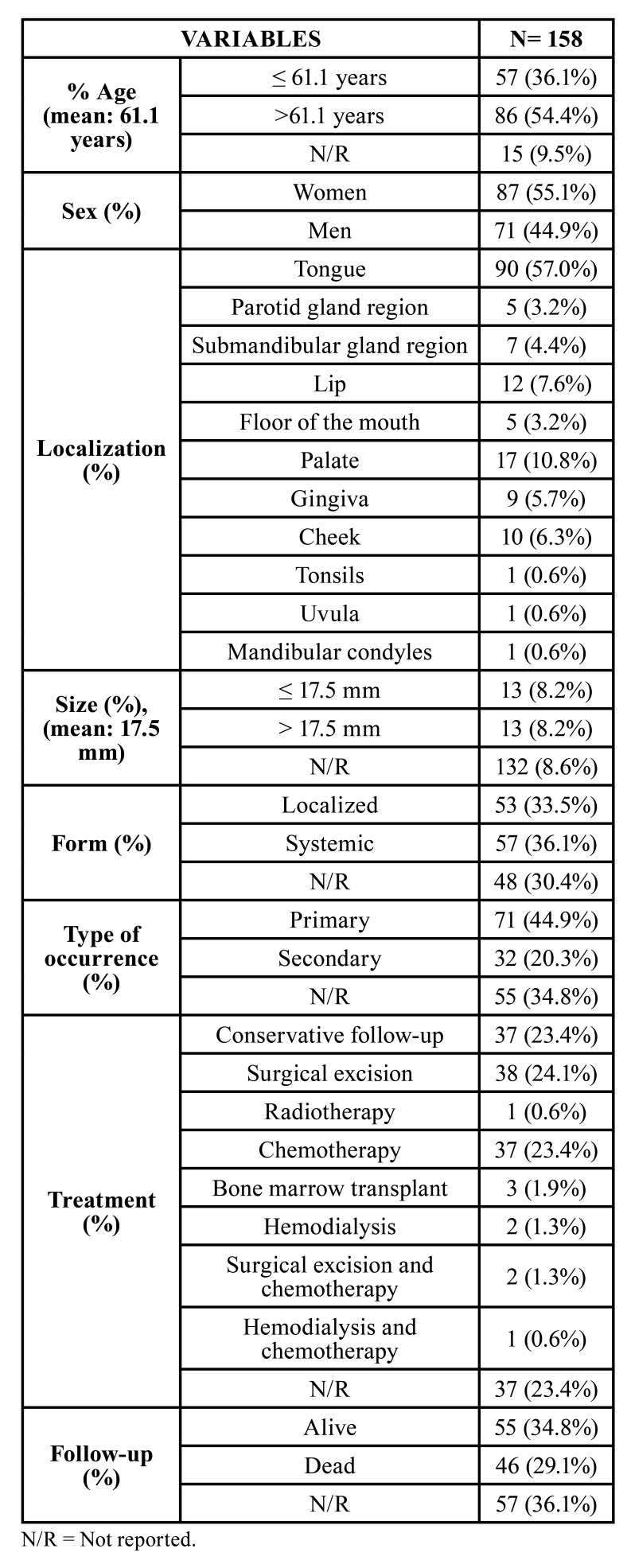
Clinical and demographic characteristics of 158 cases of Amyloidosis oral manifestations published in the literature.

Among the fifty-three cases of localized amyloidosis, the most reported symptoms were: pain/sensitivity (20.8%), dysphagia (13.2%), dysarthria (5.6%), difficulty chewing (5.6%), and weight loss (3.8%). Among the fifty-seven systemic cases, the most reported symptoms were: fatigue (36.8%), tongue swelling (36.8%), pain/tenderness (30.0%), weight loss (28.1 %), dysphagia (19.3%), xerostomia (12.3%), upper/lower limb edema (12.3%) and dysarthria (7.0%). About the locations, the most affected areas were the tongue (90 patients), followed by the palate (17 patients), lip (12 patients), buccal mucosa (10 patients), gingiva (9 patients), submandibular gland region (7 patients), parotid gland region (5 patients), floor of the mouth (5 patients), palatine tonsils (1 patient), uvula (1 patient) and mandibular condyle (1 patient). Mean lesion size was 17.5 mm (min-max: 1.8-50 mm).

In addition, on the complementary exams, the most reported imaging modality was computed tomography with 29 cases, followed by magnetic resonance with 24 cases and 12 cases underwent ultrasound. Other tests reported to assess the occurrence of systemic amyloidosis included bone marrow biopsy in 58 cases, echocardiogram in 27 cases, chest radiography in 22 cases, and electrocardiogram in 13 cases.

Regarding the clinical classification of amyloidosis, 53 cases (33.5%) corresponded to the localized form, and 57 cases (36.1%) to the systemic form. Light chain amyloidosis was reported in 39 cases, amyloid amyloidosis in 1 case, hereditary amyloidosis in 1 case, and dialysis-related amyloidosis in 7 cases. The most affected organs in the systemic form were the heart in 20 cases (35.1%), kidneys in 11 cases (19.3%), the liver in 10 cases (17.5%), skin in 9 cases (15.8 %), and gastrointestinal tract in 9 cases (15.8%). In addition, 71 cases (44.9%) corresponded to the primary form, and 32 cases (20.3%) to the secondary form. The presence of associated diseases was also analyzed, and it was observed 47 cases of multiple myeloma, 9 cases of renal failure, 7 cases of lymphomas, 7 cases of Sicca Syndrome, 4 cases of Sjogren's Syndrome and 5 cases of Carpal Tunnel Syndrome. Table 2 analyzes the relationship between the associated diseases found in the study, with the types of amyloidosis and the number of deaths of each type.

The treatment depends on the type of amyloidosis and the presence or absence of associated diseases, the most reported were conservative follow-up in 37 cases (23.4%), surgical removal in 38 cases (24.1%), radiotherapy in 1 case (0.6%), chemotherapy in 37 cases (23.4%), bone marrow transplantation in 3 cases (1.9%), hemodialysis in 2 cases (1.3%), surgical removal and chemotherapy in 2 cases (1.3%), hemodialysis and chemotherapy in 1 case (0.6%), and in 37 cases (23.4%) the treatment modality was not reported. Regarding the recurrence rate, only 4 patients (2.5%) had a recurrence in a mean period of evolution of 20 months (range 12-36 months). Patients showed a median survival time of 13.2 months (range 1-48 months). Only 46 patients died due to factors associated with the disease, most of them related to heart and/or renal failure, and 32 (88.9%) of them had systemic amyloidosis. In the other 14 cases, the type of amyloidosis was not available. Among the cases of death, 21 (58.3%) had multiple myeloma as an associated disease. Patients who died in this study from systemic amyloidosis had a mean survival time of 11.2 months.

**Table 2:**
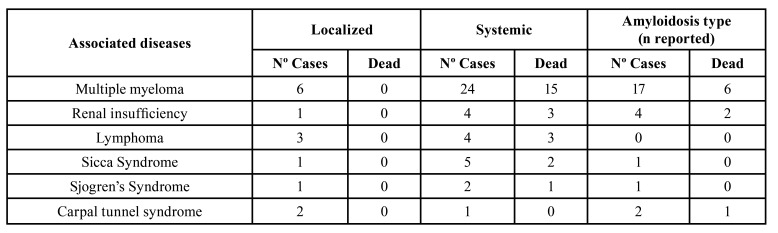
Relationship between diseases associated with oral Amyloidosis cases, type of amyloidosis and deaths in each type.

## Discussion

Amyloidosis is an uncommon group disease characterized by the extracellular deposition of amorphous fibrillar protein. This deposit can occur in any organ or tissue and depending on the amount of protein accumulated it can cause the failure of the affected organ. The first recorded use of the term “amyloid” to describe a disease was in 1842 by Rokitanki, in liver and spleen enlargement caused by chronic disease. However, it was not until 1854 that Virchow observed the presence of amylar proteins in human brains and livers (2,8,9). From these studies, several others were initiated and resulted in reports of more than 36 types of human extracellular amorphous fibrillar proteins that have been shown to be precursors of these amyloid deposits causing a variety of different diseases (1,2,8,9).

Although amyloidosis in the oral cavity is not common, when it occurs in this location, the tongue is the most affected site. However, it can also manifest in other sites such as the gingiva, lip, and palate. The clinical signs of oral amyloidosis can be macroglossia, whitish, purplish, reddish, or ulcerative nodular lesions. The most common clinical symptoms in the mouth are dysphagia, dysarthria, hoarseness, and airway obstruction may occur (10,11,12). These patterns corroborate the clinical appearances and symptoms found in this study.

Depending on the type of protein and the deposited site, amyloidosis is classified as localized or systemic. The localized form is milder, as it only affects a specific organ, as the proteins are not transported through the bloodstream (5). The systemic form affects different organs and can be divided into light chain amyloidosis (AL), amyloid amyloidosis (AA), and hereditary or familial amyloidosis (ATTR). “AL amyloidosis” is the most prevalent form and occurs due to excessive production of abnormal light chain proteins by abnormal plasma cells and seems to be the most associated with lymphoid neoplasms, including multiple myeloma. Among the 47 multiple myeloma cases included in this study in which the amyloidosis subtype was specified, 19 were AL type. The most frequently affected organs/tissues are the kidneys, liver, heart, and nerves (1).

Amyloid amyloidosis (AA) usually occurs in response to inflammatory diseases, chronic infections, or hereditary diseases such as rheumatoid arthritis and malignant tumors, including lymphomas (13,14). In addition, AA may also be present in patients undergoing daily dialysis, because of the amyloid material that is regularly deposited. All organs can be affected; however, the kidneys, liver, and spleen are the most frequent. Of the 7 patients who had some type of lymphoma in this study, 2 had AL amyloidosis and 1 had AA amyloidosis, in addition, 7 patients had dialysis-associated amyloidosis. Hereditary amyloidosis is an autosomal dominant disease caused by a mutation in the transthyretin (TTR) gene that results in the accumulation of abnormal proteins such as amyloid fibrils in various organs (9,15).

The pathologist must be aware of the clinical and histological information of the disease to be able to assess whether the deposited amyloid material corresponds to true amyloidosis, as other disorders can also cause the deposit of amyloid material, such as lipoid proteinosis, hyaline fibromatosis syndrome and, colloid milium (16). The “gold standard” for the diagnosis of amyloidosis is the use of Congo Red stain. Amyloid material stains red and displays green-yellow birefringence in polarized light. Microscopically, extracellular deposition of amorphous eosinophilic material is observed in the connective tissue, which may be arranged in the perivascular, perineural, and periadipocytic regions or diffusely throughout the tissue (Fig. 2) (12,17). It is important to know how to differentiate localized amyloidosis from systemic amyloidosis for the choice of treatment, so after the diagnosis, complementary exams are recommended to evaluate the functions of important organs, such as the heart, liver, kidneys, exams such as electrocardiogram, echocardiogram, liver, and kidney function, as well as bone marrow biopsy (5). About this, after the diagnosis of oral amyloidosis, it is necessary to investigate systemic amyloidosis and the presence of associated diseases, as some may determine the patient's survival.

The treatment will be chosen depending on the type of amyloidosis, the associated diseases, and the severity in which the organ is affected. For cases of localized amyloidosis, surgical removal of amyloid deposits is recommended. For systemic cases, chemotherapy with the use of melphalan and bone marrow transplantation are the most used treatments. Regarding the prognosis, systemic amyloidosis has a worse prognosis than the one located in the oral cavity, in addition, in the researched studies there are no indications that the localized form progresses to the systemic one. The localized form presents survival of decades, while the systemic presents an average of 20 months (1,9,18).

## Conclusions

Oral manifestations of amyloidosis presented a higher prevalence in women, with a mean age of 61.1 years old. The main affected site was the tongue, followed by the lip, and palate. The most common clinical appearances were macroglossia, purplish nodular lesions, whitish papular lesions, and ulcerated lesions. Pain, fatigue, dysphagia, dysarthria, edema, and weight loss were the most reported symptoms. The systemic form of the disease had a higher prevalence and seems to be more associated with the occurrence of multiple myeloma. In addition, chemotherapy was proved to be the treatment of choice for systemic cases and surgical removal for localized cases. The prognosis was worse for systemic cases, especially when associated with multiple myeloma.
